# Reporting behaviors and perceptions toward the National Healthcare Safety Network antimicrobial use (AU) and antimicrobial resistance (AR) modules

**DOI:** 10.1017/ice.2022.131

**Published:** 2023-03

**Authors:** Brian J. Werth, Thomas J. Dilworth, Zahra Kassamali Escobar, Alan E. Gross, Katie J. Suda, Andrew M. Morris, Jessina C. McGregor, Kristi Kuper

**Affiliations:** 1 Department of Pharmacy, University of Washington School of Pharmacy, Seattle, Washington, United States; 2 Department of Pharmacy Services, Advocate Aurora Health, Milwaukee, Wisconsin, United States; 3 Department of Pharmacy Practice, University of Illinois at Chicago College of Pharmacy, Chicago, Illinois, United States; 4 Center for Health Equity Research and Promotion, Veterans’ Affairs Pittsburgh Healthcare System, Pittsburgh, Pennsylvania, United States; 5 Department of Medicine, University of Pittsburgh School of Medicine, Pittsburgh, Pennsylvania, United States; 6 Department of Medicine, Sinai Health, University Health Network, Toronto, Ontario, Canada; 7 University of Toronto, Toronto, Ontario, Canada; 8 Department of Pharmacy Practice, Oregon State University College of Pharmacy, Portland, Oregon, United States; 9 Vizient Center for Pharmacy Practice Excellence, Irving, Texas, United States

## Abstract

**Objectives:**

To identify characteristics of US health systems and end users that report antimicrobial use and resistance (AUR) data, to determine how NHSN AUR data are used by hospitals and health systems and end users, and to identify barriers to AUR reporting.

**Design:**

An anonymous survey was sent to Society of Infectious Diseases Pharmacists (SIDP) and Society for Healthcare Epidemiology of America (SHEA) Research Network members.

**Methods:**

Data were collected via Survey Monkey from January 21 to February 21, 2020. Respondent and hospital data were analyzed using descriptive statistics.

**Results:**

We received responses from 238 individuals across 43 US states. Respondents were primarily pharmacists (84%), from urban areas, (44%), from nonprofit medical centers (81%), and from hospitals with >250 beds (72%). Also, 62% reported data to the AU module and 19% reported data to the AR module. Use of software for local AU or AR tracking was associated with increased reporting to the AU module (19% vs 64%) and the AR module (2% vs 30%) (*P* < .001 each). Only 36% of those reporting data to the AU module used NHSN AUR data analysis tools regularly and only 9% reported data to the AR module regularly. Technical challenges and time and/or salary support were the most common barriers to AUR participation cited by all respondents. Among those not reporting AUR data, increased local expectations to report and better software solutions were the most commonly identified solutions to increase AUR reporting.

**Conclusions:**

Efforts to increase AUR reporting should focus on software solutions and salary support for data-entry activities. Increasing expectations to report may incentivize local resource allocation to improve AUR reporting rates.

The Centers for Disease Control and Prevention (CDC) National Healthcare Safety Network (NHSN) has developed modules for collecting healthcare institutional data on antibiotic use (AU) and antibiotic resistance (AR) across the United States.^1^ These data can provide important, actionable feedback to antimicrobial stewardship (AMS) programs.^2^ Specifically, AUR data allow AMS programs to assess the impact of local AMS interventions and to benchmark facility-level antimicrobial consumption and resistance rates for comparison with other US facilities. Increasing the number of hospitals that report to the NHSN AUR modules would improve the quantity and richness of these national data for the benefit of US AMS programs. In a recent report, approximately one-third of hospitals enrolled in the NHSN had reported at least 1 month of AU data.^3,4^ Although the number of hospitals reporting to AUR modules is increasing, AUR reporting is not yet universal; there are technological challenges associated with AUR participation.^5^ Some facilities have encountered these or other barriers in the reporting process. To better understand the experiences of those reporting to the AU and AR modules, we surveyed members of the Society of Infectious Diseases Pharmacists (SIDP) and the Society of Healthcare Epidemiology of America (SHEA) Research Network. The survey objectives were (1) to identify characteristics of US health systems and AMS personnel who report AUR data, (2) to determine how NHSN AUR data are used by health systems, and (3) to identify barriers to AUR reporting.

## Methods

This survey was developed by infectious diseases clinicians and academics with input from stewardship practitioners and other relevant stakeholders from SIDP and SHEA. The initial draft was pilot tested by a small group of end users and was updated based on their feedback on clarity, scope, flow, content validity, and response time. The survey was reviewed by the University of Washington Institutional Review Board and was determined to be exempt from federal human-subject research regulations.

This anonymous survey was posted on Survey Monkey from January 21, 2020, to February 21, 2020, and links were e-mailed to SIDP and SHEA Research Network members (https://www.surveymonkey.com/r/WH69HT2). No exclusion criteria were applied. After the initial survey via e-mail, 2 reminder e-mails were sent: one after the first week and another after the third week. Additionally, links to the survey were posted on Twitter by SIDP after the first and third week. The branching logic survey contained 43 elements, and respondents were asked 21–35 questions, depending on their responses. The survey included questions regarding respondent and healthcare facility demographics as well as (1) use of AUR, including how and with what frequency they engage with AUR; (2) perceptions of AUR, including barriers to AUR use and potential solutions to overcome those barriers; and (3) comfort with AUR reporting. Responses were captured using a 4-point Likert scale. Potential barriers included time, salary support, information technology support, perception of benefit, institutional support, confidence in data accuracy and/or integrity, and data privacy concerns to name a few. Data from Survey Monkey were exported to Microsoft Excel (Microsoft, Redmond, WA) and were analyzed in Microsoft Excel, R Studio (R Foundation for Statistical Computing, Vienna, Austria), or GraphPad Prism software (GraphPad, San Diego, CA). Respondent and hospital data were reported as frequencies and percentages. We used the Fisher exact test to compare survey responses between 2 groups (eg, NHSN AUR reporters and nonreporters).

## Results

We received responses from 238 individuals across 43 states among 1,218 domestic SIDP and 66 SHEA Research Network members who received the survey (n = 1,284 potential participants), for an 18.5% response rate. Although 238 individuals responded to our survey, not all participants responded to all questions. Respondent demographics are summarized in Table 1 according to AU and AR data submission status. Respondents were primarily pharmacists (84%), from urban areas (44%), from nonprofit medical centers (81%), and from hospitals with >250 beds (72%). Also, 62% reported data to the AU module and 19% reported data to the AR module. The characteristics of AU and AR reporters are described in Table 2. Moreover, 43 respondents (18%) reported to both AU and AR modules, whereas 87 respondents (37%) reported to neither AU nor AR modules. Among the 118 AU and 37 AR reporters who answered questions about software use, 41% of AU reporters and 54% of AR reporters used clinical decision support software (CDSS) to compile data for upload; 54% of AU reporters and 38% of AR reporters used their electronic health record (EHR); and 5% of AU reporters and 8% of AR reporters used another method. Furthermore, 56% of AU reporters and 51% of AR reporters uploaded these data to the NHSN manually. Also, 67.8% of the 118 AU reporters and 52.6% of the 38 AR reporters answered the question about challenges reported previous or ongoing challenges to reporting to either module. Conversely, 23% and 29% responded that reporting to the AU or AR modules was easy. Specifically regarding AU reporting, common challenges included lack of information technology support (reported by 46.2%), lack of time or salary support (reported by 39.9%), and data formatting issues (reported by 33.6%). In AR reporting, common challenges included lack of information technology support (reported by 52.4%), lack of time or salary support (reported by 47.6%), and data formatting issues (reported by 37.4%). Regular use of the NHSN data analysis tools was reported by 36% of those reporting AU data and by 9% of those reporting AR data. However, 96.6% of AU reporters and 82.6% of AR reporters acknowledged some benefit to reporting, such as benchmarking or evaluating stewardship initiatives.

**Table 1.** Demographics of Those Reporting and Not Reporting to the Antibiotic Use (AU) Module and the Antibiotic Resistance (AR) Module

Characteristic	Reports AU Data, No. (%)	Does Not Report AU Data, No. (%)	Reports AR Data, No. (%)	Does Not Report AR Data, No. (%)	Total, No. (%)
**Profession**					**173 (100)**
Other	3 (1.7)	9 (5.2)	1 (0.6)	11 (6.4)	12 (6.9)
Pharmacist	96 (55.5)	49 (28.3)	36 (20.8)	109 (63)	145 (83.8)
Physician	8 (4.6)	8 (4.6)	3 (1.7)	13 (7.5)	16 (9.2)
**Facility location**					**174 (100)**
Multiple locations	17 (9.8)	9 (5.2)	8 (4.6)	18 (10.3)	26 (14.9)
Rural	16 (9.2)	13 (7.5)	5 (2.9)	24 (13.8)	29 (16.7)
Suburban	26 (14.9)	16 (9.2)	10 (5.7)	32 (18.4)	42 (24.1)
Urban	49 (28.2)	28 (16.1)	18 (10.3)	59 (33.9)	77 (44.3)
**Hospital size**					**175 (100)**
>500 beds	25 (14.3)	18 (10.3)	8 (4.6)	35 (20)	43 (24.6)
251–500 beds	29 (16.6)	16 (9.1)	16 (9.1)	29 (16.6)	45 (25.7)
101–250 beds	20 (11.4)	11 (6.3)	4 (2.3)	27 (15.4)	31 (17.7)
51–100 beds	6 (3.4)	4 (2.3)	1 (0.6)	9 (5.1)	10 (5.7)
≤50 beds	4 (2.3)	4 (2.3)	1 (0.6)	7 (4)	8 (4.6)
Multihospital system	26 (14.9)	12 (6.9)	7 (4)	31 (17.7)	38 (21.7)
**Electronic medical record**					**174 (100)**
Cerner	31 (17.8)	14 (8)	12 (6.9)	33 (19)	45 (25.9)
CPRS	7 (4)	2 (1.1)	0 (0)	9 (5.2)	9 (5.2)
Epic	52 (29.9)	39 (22.4)	18 (10.3)	73 (42)	91 (52.3)
Meditech	14 (8)	3 (1.7)	4 (2.3)	13 (7.5)	17 (9.8)
Other	5 (2.9)	7 (4)	1 (0.6)	11 (6.3)	12 (6.9)
**Hospital type**					**174 (100)**
For profit	15 (8.6)	3 (1.7)	5 (2.9)	13 (7.5)	18 (10.3)
Government	5 (2.9)	5 (2.9)	1 (0.6)	9 (5.2)	10 (5.7)
Not for profit	86 (49.4)	55 (31.6)	31 (17.8)	110 (63.2)	141 (81)
Veterans’ Affairs	4 (2.3)	1 (0.6)	0 (0)	5 (2.9)	5 (2.9)
**Teaching affiliation^a^**					**175 (100)**
Nonteaching facility	28 (16)	12 (6.9)	9 (5.1)	31 (17.7)	40 (22.9)
Medical school	43 (24.6)	35 (20)	16 (9.1)	62 (35.4)	78 (44.6)
Medical residency	72 (41.1)	45 (25.7)	26 (14.9)	91 (52)	117 (66.9)
Pharmacy school	39 (22.3)	31 (17.7)	15 (8.6)	55 (31.4)	70 (40)
Pharmacy residency	64 (36.6)	47 (26.9)	19 (10.9)	92 (52.6)	111 (63.4)
Nursing school	38 (21.7)	35 (20)	16 (9.1)	57 (32.6)	73 (41.7)
Allied health professions	40 (22.9)	26 (14.9)	16 (9.1)	50 (28.6)	66 (37.7)
**Software for Tracking Local Antimicrobial Use and Resistance Data (Non-NHSN)^a^**					**238 (100)**
Other	42 (17.6)	34 (14.3)	11 (4.6)	65 (27.3)	76 (31.9)
Vigilanz	15 (6.3)	3 (1.3)	12 (5)	6 (2.5)	18 (7.6)
Cerner	19 (8)	14 (5.9)	6 (2.5)	27 (11.3)	33 (13.9)
None	4 (1.7)	17 (7.1)	0 (0)	21 (8.8)	21 (8.8)
Theradoc–Premier	34 (14.3)	16 (6.7)	9 (3.8)	41 (17.2)	50 (21)
Epic	64 (26.9)	31 (13)	18 (7.6)	77 (32.4)	95 (39.9)

Note. CPRS, computerized patient record system; NHSN, National Health Safety Network.

a Percentages do not add to 100% due to multiple selections.

**Table 2.** Characteristics of Those Reporting to the Antibiotic Use (AU) Module and the Antibiotic Resistance (AR) Module

Question and Responses	Reports AU Data, No. (%)	Reports AR Data, No. (%)
**Does sending your Antimicrobial Use (AU) data to NHSN require a manual upload or is it directly sent from your software vendor?**	N = 148	N = 46
Direct	50 (33.8)	19 (41.3)
Manual	64 (43.2)	19 (41.3)
No answer	34 (23)	8 (17.4)
**Who is responsible for AU or AR reporting?**	N = 152	N = 49
Data analyst or information technology specialist	20 (13.2)	5 (10.2)
Infection prevention specialist	35 (23)	17 (34.7)
Microbiology/clinical laboratory specialist	0 (0)	5 (10.2)
Pharmacist	83 (54.6)	21 (42.9)
Physician	7 (4.6)	1 (2)
Other or don’t know	7 (4.6)	0 (0)
**Person responsible for reporting is the same as NHSN infection reporting**	N = 148	N = 46
No	74 (50)	25 (54.3)
Yes	42 (28.4)	16 (34.8)
Unknown or no answer	32 (21.6)	5 (10.9)
**How often do you report to NHSN AU or AR?**	N = 149	N = 46
Every 6 months	1 (0.7)	1 (2.2)
Every month	100 (67.1)	26 (56.5)
Every quarter	14 (9.4)	8 (17.4)
N/A	30 (20.1)	7 (15.2)
Unknown	4 (2.7)	4 (8.7)
**What has been your experience with reporting to AU or AR?**	N = 118	N = 38
Challenging to get started	60 (50.8)	13 (34.2)
Easy	27 (22.9)	11 (28.9)
Someone else reports.	11 (9.3)	7 (18.4)
Still challenging	20 (16.9)	7 (18.4)
**How often do you use the data analysis tools available in NHSN?**	N = 117	N = 38
I have never used these tools.	30 (25.6)	21 (55.3)
I have used these tools a few times but not regularly.	33 (28.2)	13 (34.2)
I use these tools at least monthly.	19 (16.2)	1 (2.6)
I use these tools at least quarterly.	35 (29.9)	3 (7.9)
**Which of the following do you consider to be benefits of participating in AUR reporting?**	N = 118	N = 39
Useful for benchmarking our institutional antibiotic consumption to similar hospitals and to national trends	104 (88.1)	N/A
Useful for evaluating stewardship initiatives	82 (69.5)	18 (46.2)
Generates data that justify my position	29 (24.6)	8 (20.5)
Demonstrates the value of ASP to local stakeholders	51 (43.2)	10 (25.6)
Responsibility that offers global benefit for antimicrobial resistance tracking	54 (45.8)	22 (56.4)
Creates financial gains for my institution	9 (7.6)	2 (5.1)
There are no direct benefits to health systems that report NHSN AUR data.	5 (4.2)	8 (20.5)

Note. ASP, antimicrobial stewardship program; AUR, antibiotic use and resistance; NHSN, National Health Safety Network.

The characteristics and perceptions of AU and AR nonreporters are summarized in Table 3. Among nonreporters, increased institutional pressure to report and local availability of software that streamlines data compilation and submission were the most commonly identified means to increase reporting. The most common barriers to reporting to either the AU or AR modules were related to lack of dedicated information technology support, lack of time or salary support, and data formatting issues. Very few respondents (1.2%) expressed concerns about data privacy or security issues, which accounted for 0.5% of barriers reported. When asked about their experiences, 40% of respondents not reporting to the AU module said that they planned to begin within the next year, compared to only 25% of those not reporting to the AR module. Among nonreporters to AU and AR respectively, 15% and 10% indicated that they were unable to report, and 12% and 5% tried reporting but encountered barriers. Furthermore, >30% of AU nonreporters and >50% of AR nonreporters had not attempted reporting. Only a small number of respondents cited a need for more or better AUR module training than what is currently available to end users from the CDC.^6–8^

**Table 3.** Perceptions Among Those Who Do Not Report Data to the Antibiotic Use (AU) Module and the Antibiotic Resistance (AR) Module

Question and Responses	Does Not Report AU Data, No. (%)	Does Not Report AR Data, No. (%)
**What has been your experience with reporting to AU or AR?**	N = 91	N = 141
Not applicable	8 (8.8)	10 (7.1)
We have decided to not report our data.	2 (2.2)	15 (10.6)
We have not considered reporting at our institution(s).	8 (8.8)	36 (25.5)
We have tried to report but have given up or encountered barriers.	10 (11.0)	6 (4.3)
We plan to begin reporting in the next 6–12 months.	34 (37.4)	34 (24.1)
We want to report but we are currently unable.	13 (14.3)	14 (9.9)
We want to report but we have not gotten around to trying it yet.	16 (17.6)	26 (18.4)
**Which of the following have the greatest impact on increasing AU or AR reporting?**	N = 81	N = 132
Increased pressure to report from my institution, the CDC, or professional organizations	22 (27.2)	35 (26.5)
Position dedicated to supporting AU reporting	11 (13.6)	18 (13.6)
More comprehensive data collection to improve the usefulness of the resulting dataset	6 (7.4)	12 (9.1)
Online training materials beyond those currently available	3 (3.7)	3 (2.1)
Partial pharmacy position dedicated to supporting AU reporting	11 (13.6)	15 (11.4)
Software vendor providing solutions to streamline the compilation of data in appropriate formats	28 (34.6)	49 (37.1)

Note. CDC, Centers for Disease Control and Prevention.

Respondents already using software for local AU or AR tracking were more likely than those not using software for local tracking to report to the AU module (19% vs 64%) and the AR module (2% vs 30%; *P* < .001 for both).

## Discussion

Measuring antimicrobial consumption and antibiotic resistance locally are indispensable to track and tailor AMS programs and are recommended by the CDC core elements of antimicrobial stewardship.^9^ In addition, the NHSN AUR module aggregates data to create benchmarks and to guide a national AMS strategy.^1^ Importantly, national data from the CDC Emerging Infection Program were used to help establish national targets to reduce inappropriate antibiotic prescribing.^10,11^ The number of healthcare facilities participating in AUR reporting is increasing. Our results suggest that participation in AU reporting may continue to increase; nearly half of survey respondents not reporting AU data stated that they planned to start submitting data to NHSN AU within the next year. However, only 25% of respondents stated that they were submitting AR data to the NHSN, and of those not reporting AR data, only 25% intended to do so in the next year. This finding may be related to differential access to pharmacotherapeutic data compared with infection prevention and microbiology data by pharmacists, which accounted for 84% of our survey respondents. In our study, a plurality of respondents indicated that pharmacists were responsible for reporting AR data at their institutions, even though AR data are outside the domain of pharmacy information. Collaboration between AMS clinicians and local clinical microbiology and infection prevention partners might facilitate greater participation in AR reporting, or perhaps AR reporting duties should be delegated to the departments that maintain these data. Nevertheless, increasing participation in the NHSN AUR module could advance AMS practices. Trends in antimicrobial resistance, compared to antimicrobial use, are a clearer representation of the importance and the impact of antimicrobial stewardship both among practicing clinicians and the communities in which their institutions reside. In our small study, we were not able to identify significant differences among health systems that report or do not report to the AUR modules with confidence. However, we did uncover 2 main opportunities to increase NHSN AUR participation: information technology or software solutions and increasing local submission expectations or incentives.

Improved information technology for enhancing participation in AUR reporting is somewhat self-evident and has a clear solution. The easier it is for AMS clinicians to submit their data, the more likely they will be to do so. The CDC endorses systematic and standardized AUR data collection practices while advocating for the automation of data submission to minimize time spent on reporting by AMS clinicians.^1^ However, technological challenges to AUR participation have been well described.^4^ Given the numerous EHR systems in use within the United States (Table 1), it is not surprising that information technology or software issues were cited by respondents as the largest barrier to NHSN AUR submission. Interestingly, we observed differences in the number of respondents submitting to AUR modules based on the EHR or CDSS used at their facility. Certain EHR or CDSS platforms may interface better with the AUR module than others. It will be important for CDC, AMS clinicians, EHR and CDSS vendors, and other stakeholders to ascertain which aspects of certain EHR or CDSS platforms facilitate better AUR data submission. Very few respondents had concerns with data privacy related to AUR data submission. More than half of those reporting to the AU or AR module manually scheduled their clinical data architecture file (CDA) file uploads, and others used software that automatically compiled and uploaded these files, reducing workload. Manual scheduling of these file uploads adds to the workload for AMS clinicians and may create data integrity issues; thus, the CDC advocates direct data submission.^1^ EHR and CDSS software companies and the CDC must collaborate on solutions that allow all AMS clinicians in the United States to compile and submit data to the AUR modules directly. This private–public sector collaboration, or additional federal funding to support the NHSN, would reduce the reporting burden on an already strained AMS workforce and would increase the quantity, richness, and generalizability of AUR data.

The latter opportunity, increasing local submission expectations, is less straightforward. Respondents indicated that added institutional pressure to submit data to the AUR modules would increase their likelihood of submission. Many reported low institutional priority for AUR data submission; particularly for AR data. Thus, increased institutional and organizational expectations for AUR data submission may be needed to increase reporting. Our findings indicate that the implementation of these efforts would be sufficient for many AMS clinicians not currently submitting AUR data. These clinicians are surely advocating for AUR submission at their facility; many respondents reported trying to submit data but then giving up due to submission barriers. A corollary is that the voluntary reporting of medical errors leaves unknown the true number of medical errors in the United States.^12^ Efforts are needed by the CDC and other healthcare regulatory bodies (eg, The Joint Commission) that adjudicate whether facilities meet AMS standards to incentivize institutions to submit AUR data. Or perhaps regulatory bodies should require AUR data submission in their AMS standards. The adoption of the National Quality Forum (NQF)–endorsed standardized antimicrobial administration ratio (SAAR) metric from the CDC as a required benchmark for hospitals under the Centers for Medicare & Medicaid Services conditions of participation would likely increase reporting substantially. However, the use of the SAAR as a neutral benchmark and not a specific goal would need to be emphasized, given that this metric does not adjudicate antibiotic appropriateness. Without a multipronged approach to incentivizing healthcare institutions and their leaders to prioritize AUR data submission, it is unlikely to happen. Notably, currently pending legislation may help prioritize AUR data submission for healthcare systems. The DISARM and PASTEUR acts aim to reimburse higher-cost antimicrobials outside the capitated diagnosis-related group (DRG) payment. However, this legislation includes a requirement for eligible hospitals to submit their AUR data to the NHSN.^13^ Healthcare facilities can also exert pressure on EHR software providers to help develop solutions for AUR data submission.

A final opportunity may be to increase either the utility or accessibility of AUR analytics tools. Only ∼20% of respondents thought that making the AUR data more useful was an important barrier to AUR reporting. However, regular AUR tool use in the NHSN was reported by only 36% and 9% of those reporting AU and AR data, respectively, indicating that most users have never or have only rarely used these tools. We did not survey respondents in detail regarding their use of these analytics tools, so the best intervention to increase the use of AUR tools remains unclear. But if users valued the tools highly, then they would have greater incentive to report. Perhaps redesigning these analytic tools and/or promoting their utility to current and potential reporters would increase the number of facilities reporting AUR data. One example that surfaced in discussion with current users is that the antibiogram in the NHSN reports shows the percentage resistant rather than the percentage susceptible, which, is an uncommon style of antibiogram reporting and requires transformation to adapt to end-user purposes.

Many of the barriers reported in this survey are outside the control of AMS clinicians. This lack of agency toward something AMS clinicians wish to do may have led to the unfavorable sentiment that many respondents felt toward AUR data submission. Interestingly, those not reporting AU data were less likely to think that their colleagues encountered barriers than those who are active reporters. Our results also indicate that productive conversations and resource sharing among AMS colleagues about NHSN AUR reporting are relatively uncommon.

Our survey is the first to describe AMS clinician use of, perceptions of, and comfort with the NHSN AUR module. Our results offer insights into end-users’ understanding of the AUR modules across a diverse cross section of US health systems, and we have identified clear opportunities to increase the number of facilities that submit data to the AUR modules. More complete AUR data could help propel AMS research, but implementation efforts are needed.

This study had several limitations. The survey had a low response rate, and the proportion of AU responders was much higher than that of AR responders or nonresponders, specifically pharmacists. Our results are mainly descriptive results, based on a small cohort of potential AUR users in the United States. Because we only sampled hospitals affiliated with an SIDP member of the SHEA Research Network, hospitals with less antimicrobial stewardship resources and staff, including critical access and/or rural hospitals, which are probably unlikely to be submitting antibiotic data to the AUR, were likely underrepresented in this study. Despite oversampling those familiar with the intent of AUR and the motivation to submit, we detected substantial discontent and/or disinterest in submitting data to the AUR modules, largely because of the challenges with submission and the perceived limited utility of the data and its tools. Since our survey was conducted, some of the updates to NHSN AUR data visualization tools may have improved the user experience or usefulness of the tools. Thus, future surveys should attempt to secure AUR feedback from all possible users to reflect changes to the platforms. Additionally, future AUR research should include capturing end-user feedback, enhancing adoption of software solutions, and facilitating coordination between end users, healthcare regulatory bodies, and the CDC to set expectations for AUR data submissions. Routine CDC AU and AR module user calls, which create a forum to share experiences between facilities using the product and capture end-user feedback, are in place; however, the perception of respondents was that these opportunities are not used enough and could be bolstered.

In conclusion, our survey results reveal opportunities to increase the number of facilities reporting AUR data to the NHSN. Efforts should focus on finding software solutions and increasing institutional expectations to report these data. Increasing the utility of AUR analytical tools may also incentivize more institutions to participate. The lower rates of AR reporting compared to AU reporting may suggest a need for future interventions specifically targeting the AR module.
